# Family Poultry Farming in Sri Lanka: An Integral Component of Protein Security, Poverty Alleviation and Public Health

**DOI:** 10.1002/vms3.70214

**Published:** 2025-01-15

**Authors:** Umayangana Pujani Gunasekara, Anil Wasantha Kalupahana, Yasodhara Deepachandi Gunasekara, Ayona Silva‐Fletcher, Ruwani Sagarika Kalupahana

**Affiliations:** ^1^ Faculty of Veterinary Medicine and Animal Science University of Peradeniya Peradeniya Central Sri Lanka; ^2^ Asia‐Pacific Centre for Animal Health Melbourne Veterinary School, University of Melbourne Victoria Australia; ^3^ The Royal Veterinary College University of London London UK

**Keywords:** disease transmission, farmer education, poultry disease, vaccination awareness

## Abstract

Family poultry farming plays a crucial role in ensuring protein availability and household income, particularly in low‐income families. This study investigates the knowledge, attitudes and practices of family poultry farmers regarding poultry diseases, biosecurity and vaccinations. The research involved surveying 150 farmers in a selected area in Sri Lanka's western province, revealing significant knowledge gaps, particularly in understanding poultry diseases, their causes, transmission pathways and potential impacts on humans. Most respondents demonstrated a lack of familiarity with biosecurity practices, with only a small fraction accurately identified recommended measures. Among the total respondents, only 16.7% were familiar with the term ‘biosecurity’. Additionally, only 29.3% recognized that family poultry could pose a threat to public health. A significant majority (84.67%) admitted to not knowing which diseases could be effectively prevented through vaccination, and 80% of respondents had never vaccinated their birds. These findings highlight the challenges faced by family poultry farmers due to their limited understanding of crucial aspects of poultry management. The study underscores the need for government intervention and policy development to address these issues. Education and training programmes are essential to enhance farmers’ awareness regarding disease control, biosecurity measures and the benefits of vaccination.

## Graphical abstract

This study reveals significant knowledge gaps among family poultry farmers in Sri Lanka regarding poultry diseases, biosecurity and vaccinations. Among the participants, 85% did not know which diseases could be prevented through vaccination. The findings underscore the need for targeted education and government intervention to improve disease management practices

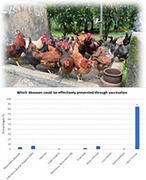

## 1 Introduction

Family poultry farming occupies a pivotal role in global poultry production systems (Lal et al. 2021). It serves as a potent tool in the battle against protein malnutrition (Rajkumar et al. 2021). This is especially pertinent for underprivileged households, who can meet their daily protein needs through domestic family poultry farming initiatives. Moreover, family poultry farming is increasingly acknowledged as an effective means of income generation for economically disadvantaged communities, concurrently elevating their purchasing power. This practice also contributes to the empowerment of women in diverse regions worldwide (Kumar, Dahiya, and Ratwan 2021). In India, backyard family poultry farming significantly contributes to egg production and offers poverty alleviation and rural employment benefits, particularly for women and tribal areas (Rajkumar et al. 2021). The global poultry industry, particularly intensive commercial operations, encountered substantial setbacks during the COVID‐19 pandemic. Scarcities in medicines, feeds and labour exposed vulnerabilities in this sector. Consequently, the significance of family poultry farming has surged as an alternative to commercial poultry operations due to its comparatively lower dependence on commercial poultry feeds, medicines and labour forces (Das and Samanta 2021). Similarly, the poultry industry in Sri Lanka faced substantial disruptions due to the COVID‐19 pandemic and subsequent economic downturn. An ongoing study investigating the impacts of pandemic and economic downturn on poultry industry has identified the factors that lead to the disruption and indicates the less impact on family poultry where the farms are not highly dependent on commercial feed. Less labour cost, market potential to sell excess production and lower production costs have been identified as benefits of village chicken egg production (Jayasooriya et al. 2023).

Family poultry, typically raised in free‐range settings, confront unique challenges such as exposure to predatory animals, wild birds and other livestock (Estifanos and Tadesse 2021). Suboptimal management practices, primarily stemming from predation and infectious diseases, are often observed within family poultry operations. Native poultry breeds are commonly favoured for backyard farming due to their relatively good resistance to endemic diseases (Singh et al. 2021). However, they can inadvertently act as reservoirs for pathogens, posing a potential threat to the commercial poultry reared in proximity (Ahmed, Ameer, and Javed 2021). Additionally, their close interaction with households raises concerns about public health, as there is a risk of exposure to zoonotic diseases (Keerthirathne et al. 2022).

Studies on backyard poultry farm management have been reported worldwide. These studies explore poultry farmers' practices, knowledge, attitudes, and the socio‐cultural and economic factors related to backyard poultry farming. In Bangladesh, it is reported that 34% of backyard chickens rely on scavenging for feed (Khalil et al. 2023). Backyard poultry management practices in Kenya showed that farmers use antibiotics for prophylactic treatments, as growth promoters, and for the purpose of increasing egg production (Kiambi et al. 2021). Commercial poultry farmers in Nepal were aware of avian influenza, but will not report outbreaks due to the fear of poultry being culled and inadequate compensation (Lambrou et al. 2020). Framer educational levels were linked to knowledge regarding zoonosis; those with higher education levels correlated with better prevention perceptions (Niraulaa, Sharmaa, and Dahalb 2021). However, knowledge is not necessarily linked to practices. Backyard poultry farmers in Nigeria also showed that adequate farmer knowledge of chicken coccidiosis, but did not use of vaccines as a preventive measure (Adeyemi et al. 2023). Marketing their products is another challenge for family poultry keepers. Lack of access to roads and markets was identified as a major challenge for rural farmers in Kenya (Chaiban et al. 2020).

The poultry industry in Sri Lanka has risen in importance, making a significant contribution to the national gross domestic product. Similar to many other developing nations, family poultry farming plays a pivotal role in reducing protein malnutrition and empowering low‐income households thereby adding to food security, an essential sustainable development goal. In Sri Lanka, village chicken egg production has emerged as a significant sector. In Sri Lanka, the total number of egg production was 2934.55 million in 2021 (Department of Animal Production and Health, n.d.). The total number of layer birds in 2021 was 7,039,930, out of which 1,160,740 were village chickens. Further, percentage of layer village chicken was 16.48% in 2021 (Department of Census and Statistics, n.d.). Also, a research conducted in Anuradhapura revealed that the average flock size of village chickens was 30.09. Further, it revealed that the average egg consumption of the owners of the flocks was 81 eggs per month, and the average monthly sale was 255 eggs (Jayasooriya et al. 2023). In 2024, the selling price of egg fluctuated around 55–65 rupees, providing a good income source for farmers. Besides, the premium price for village chicken eggs is significantly high in Sri Lanka. Less labour cost, the market potential to sell excess production and lower production costs have been identified as the main benefits of village chicken egg production (Jayasooriya et al. 2023). Generally, village chicken eggs command a premium price over regular table eggs in Sri Lanka. Additionally, there is a common belief among the public that village chicken eggs are more nutritious than regular table eggs, influencing consumer preferences (Ariyachandra et al. 2023).

The poultry industry in Sri Lanka faced substantial disruptions due to the COVID‐19 pandemic and subsequent economic downturn. An ongoing study is looking to the impacts of pandemic and economic downturn on poultry industry. It has clearly identified the factors that led to the disruption and indicates less impact on family poultry where the farms are not highly dependent on commercial feed. Less labour costs, market potential to sell excess production and lower production costs have been identified as benefits of village chicken egg production (Jayasooriya et al. 2023).

Despite the timely need for its expansion, the management practices of family poultry in Sri Lanka remain inadequately documented. Moreover, comprehending the scale of family poultry operations within the country and assessing their biosecurity measures is crucial for managing disease risks to the general public and the commercial poultry sector (De Zoysa et al. 2022). Therefore, mapping the family poultry network would enable the identification of possible hazards and the establishment of critical control points to mitigate these threats. In addition, assessment of the weaknesses and the potentials to improve would be beneficial to upgrade the family poultry.

This research constitutes the preliminary phase of a broader project aimed at developing evidence‐based policies related to family poultry. Exploring the knowledge, attitude, and practices (KAP) among family poultry farmers is essential for establishing a sustainable family poultry farming system in Sri Lanka. Surprisingly, to date, such comprehensive studies have not been reported in Sri Lanka. Thus, this study was designed to survey and document the KAP concerning management and biosecurity practices among family poultry keepers in the Western Province of Sri Lanka. Understanding the knowledge gaps and needs of family poultry farmers will enable the development of training programmes and educational materials that better align with their requirements.

### 1.1 Theoretical Foundation for the Study

The current study employs the KAP theoretical framework, which provides a structured approach to assess the current state of family poultry farming by examining what farmers know about biosecurity and disease prevention (Knowledge), their perceptions and beliefs about these practices (Attitudes), and the actual implementation of these practices (Practices) (Andrade et al. 2020). The KAP framework is particularly valuable for its ability to identify discrepancies between knowledge, attitudes and practices, thus highlighting specific areas where educational interventions and behaviour change strategies are needed. This framework is instrumental in designing targeted interventions that are culturally and contextually relevant, ensuring that they effectively address the unique challenges faced by family poultry farmers in Sri Lanka. When compared to other frameworks, such as the Health Belief Model (HBM), Socio‐Cognitive Theory (SCT) or COM‐B model, the KAP framework offers distinct advantages. The HBM focuses primarily on individual perceptions of health threats and the benefits of preventive actions, which can be limiting when addressing complex agricultural practices that are influenced by broader socio‐economic and environmental factors (Champion and Skinner 2008). HBM's utility is further constrained by its limited scope in understanding the depth of knowledge or the complexity of attitudes towards biosecurity and management practices in family poultry farming. The SCT emphasizes the role of observational learning, self‐efficacy and environmental influences on behaviour. While SCT provides a comprehensive understanding of behaviour change mechanisms, its application requires extensive resources and ongoing support, which may not be feasible in resource‐constrained settings typical of rural Sri Lankan communities (Heffernan 1988). The KAP framework, with its straightforward methodology and focus on the triad of KAP, provides a pragmatic approach that is both cost‐effective and easy to implement. It facilitates the identification of key areas for intervention, enabling the development of tailored educational programmes and practical demonstrations that can significantly improve biosecurity and management practices among family poultry farmers. For instance, by understanding the specific knowledge gaps and misconceptions that farmers hold, educational interventions can be precisely targeted to address these issues (Makita et al. 2020). Similarly, by gauging farmers' attitudes, it is possible to identify motivational barriers or facilitators that could influence the adoption of improved practices. Moreover, the KAP framework's adaptability allows for the integration of qualitative insights with quantitative data, providing a richer, more nuanced understanding of the farmers' behaviours and the socio‐cultural context within which they operate (Wan, Rav‐Marathe, and Marathe 2016). This comprehensive perspective is crucial for designing interventions that are not only effective but also sustainable and culturally appropriate. By integrating the KAP framework into this study, we aim to generate actionable insights that can enhance the sustainability and resilience of family poultry farming in Sri Lanka, ultimately contributing to improved food security and economic well‐being in rural communities. The insights derived from this framework will inform policy recommendations, capacity‐building initiatives, and community‐based programmes aimed at empowering farmers with the knowledge and resources they need to implement effective biosecurity measures and optimize their poultry management practices.

## 2 Methods

### 2.1 Study Area

The research was conducted in the Western Province of Sri Lanka, situated in the southwestern part of the country. This province encompasses an area of 3684 square kilometres (1422 square miles) and comprises three districts: Colombo (Latitude: 6.9271°N, Longitude: 79.8612°E), Kalutara (Latitude: 6.5836°N Longitude: 79.9595°E) and Gampaha (Latitude: 7.0912°N, Longitude: 79.9944°E). The Western Province was purposively selected due to its convenience and the high concentration of family poultry production. The study encompassed all three districts within the Western Province of Sri Lanka, with data collection taking place from January to March 2022.

Data collection was facilitated through the use of a self‐designed, structured questionnaire. This tool aimed to capture information regarding the socio‐demographic characteristics of participants and their KAP related to the management and bio‐security of family poultry in the Western Province. Multiple‐choice questions and Likert scale questions were included in the questionnaire. The multiple‐choice questions aimed to gather specific information on various aspects of family poultry farming practices, while the Likert scale questions assessed the respondents' understanding regarding management and biosecurity practices. The questionnaire was initially developed in English by subject matter experts to ensure its content and relevance, and it was subsequently translated into the local (Sinhala) language. Before its final distribution, the questionnaire underwent a pilot trial involving 20 family poultry farmers in the selected area. The questionnaire was revised and finalized based on the results of the pilot study.

### 2.2 Sampling

The administration of 150 researcher‐led survey questionnaires was guided by our funding allocation, which served as a foundational guideline for our sample size. The absence of an accurate register of family poultry farmers in the study areas compelled us to adopt a pragmatic approach to sample selection.

A multi‐stage random sampling approach was employed. In Stage 1, the study began by selecting the Western Province out of the nine provinces in Sri Lanka due to the high concentration of family poultry production (Department of Animal Production and Health, n.d.). This province comprised three districts: Colombo, Gampaha and Kalutara. Stage 2 involved the random selection of Divisional Secretariat (DS) divisions from each district: Padukka for Colombo, Divulapitiya for Gampaha, and Alagawatta and Palinda Nuwara for Kalutara. In Stage 3, within each DS division, five Grama Niladhari (GN) divisions were randomly selected. Stage 4 utilized the non‐probability sampling technique of snowball sampling to select 10 family poultry farmers from each GN division.

Local government field officers, such as Samurdhi development officers and development officers, who were familiar with the selected area, assisted in locating family poultry farmers. Only family poultry farmers who maintained five or more poultry birds were included in the survey. The questionnaires were conducted with the individual primarily responsible for family poultry rearing in each household, and if that individual was unavailable, a second responsible person within the same household was interviewed.

### 2.3 Data Analysis

Descriptive statistics such as charts, percent and frequency were used to summarize the data, with total numbers and percentages calculated for various variables. Respondents who adhered to five or six of the six biosecurity measures were categorized as ‘good’. Those who followed four or three measures were categorized as ‘moderate’, and those who followed only one or two were categorized as ‘poor’.

## 3 Results

### 3.1 Demographic Characteristics of the Participants

The demographic profile of the respondents in the survey provides valuable insights. A substantial majority of respondents were female, constituting 68% of the total. Approximately 65% of respondents had completed their education up to the General Certificate of Education—Ordinary Level (G.C.E. O/L) which is equivalent to 11 years of school education. The survey revealed that the majority of respondents had relatively small families, with 81% reporting five or fewer family members. In terms of experience in family poultry farming, the average years of experience in family poultry is approximately 10 years (Table 1).

**TABLE 1:** Demographics of respondents in relation to the gender, age, religion, education qualification, total number of family members and experience in family poultry farming (years).

Characteristics	Range/group	*n*	%
Sex	Male	48	32
	Female	102	68
Age	< 18	3	2
	18–40	49	32.67
	41–60	79	52.67
	> 60	18	12
Religion	Buddhist	95	63.3
	Hindu	27	18
	Catholic/Christian	11	7.3
	Islam	17	11.3
Highest education qualification	Primary	30	20
	Up to O/L	97	64.7
	Up to A/L	15	10
	Diploma	1	0.7
	Degree	1	0.7
	Never had formal school education	6	4
Total number of members in the family	5 or below	121	80.6
	> 5	29	19.3
Experiences in family poultry farming (years)	< 1	19	12.66
	1–10 years	80	53.3
	11–20 years	20	13.33
	> 20	31	20.6
			

### 3.2 Characteristics of the Studied Farms

The majority of respondents (60%) reported having a flock size of 5–15 birds, while 34% had 16–30 birds, and a smaller proportion (6%) had more than 30 birds. Also, 90% of respondents primarily focused on egg production, 2% on meat production and 7% engaged in both egg and meat production. Only one person (1%) reared family poultry as a pet animal. Around 33% raised poultry solely for family consumption, while 64% aimed to generate additional income for their families, and 3% considered poultry farming their main income source.

Among studied farms, 47% kept chickens inside pens at all times. In 51% of the farms, pens served as night‐time housing, while free‐ranging was permitted during the daytime. Conversely, 3% of the farms practiced free‐range rearing throughout the entire day. Approximately 49% had companion animals, 27% kept livestock and 13% raised other bird species such as parrots and pigeons as pet animals alongside chicken.

Also, 34% of respondents personally carried out all the farm work themselves, while 66% obtained the support of their family members in the family poultry farming. Moreover, 63% dedicated 1 h or less, and 29% spent more than 1 h/day on poultry farming. Among these farmers, half of the farmers sold products to neighbours and friends, 14% to neighbouring shops, 4% owned a farm shop to sell their products and 4% sold their poultry products to middlemen.

Ground wells were the primary water source (80%), while 20% relied on pipe‐borne water. Most farmers (57%) fed chickens both commercial feed and leftover family food, 35% allowed chickens to scavenge and 20% provided only commercial feed.

### 3.3 KAP Regarding Biosecurity, Poultry Diseases and Vaccinations

#### 3.3.1 Awareness of Biosecurity

**FIGURE 1:**
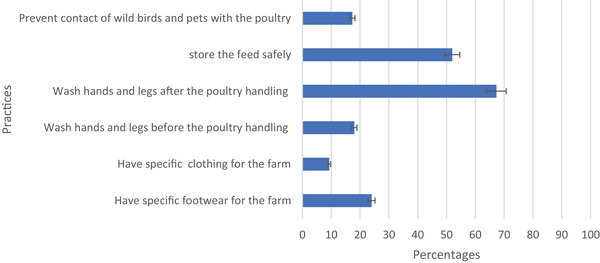
Biosecurity practices followed by respondents.

The study investigated the common basic biosecurity practices followed by the respondents, focusing on six key practices (Figure 1). The most common biosecurity measure selected was ‘Washing hands and legs after poultry handling’, with 67% of respondents recognizing its importance. In contrast, ‘Having specific clothing for the farm’ had the lowest recognition, with only 9% of respondents selecting this measure. However, among the total population of respondents, 25 (17%) were familiar with the term ‘biosecurity’, with the vast majority (83%) indicated their lack of familiarity.

**FIGURE 2:**
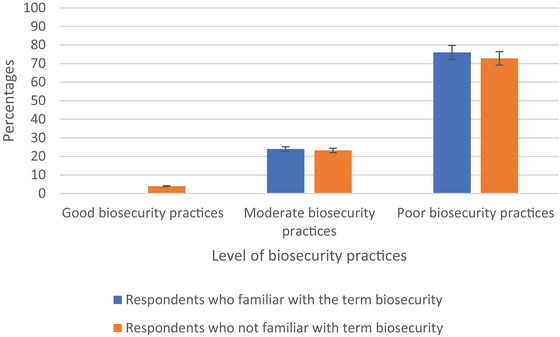
Levels of biosecurity practices respected according to their familiarity with the term ‘biosecurity’.

Among the respondents familiar with the term ‘biosecurity’, none demonstrated good biosecurity practices. The majority, 76%, exhibited poor biosecurity practices. In contrast, among respondents not familiar with the term ‘biosecurity’, 73% had poor biosecurity practices (Figure 2).

#### 3.3.2 Awareness of Poultry Pathogens and Disease Transmission

When requested to identify causes of poultry diseases, approximately 21% recognized viruses as a significant cause, while 11% attributed diseases to bacteria. Parasites and poor environment were less commonly mentioned, at 3% and 7%, respectively. Notably, 57% admitted to have no specific knowledge about causes of poultry diseases

In response to whether disease‐causing microorganisms could spread among chickens, 54 believed they could, 19 thought they could not, and 77 were uncertain. The analysis of responses regarding the spread of disease‐causing microorganisms among chickens reveals several key findings. The average opinion among respondents is approximately 2.26, indicating a tendency towards uncertainty, with a standard deviation of approximately 0.731, suggesting moderate variability in opinions. Furthermore, the coefficient of concordance (Kendall's W) is approximately 0.189, indicating a relatively low level of agreement among respondents' opinions.

Respondents identified various sources of disease transmission to poultry. Approximately 21% pointed to feed, 29% to water and a smaller percentage cited wild animals (5%) and companion animals (5%). Around 22% identified feed or water utensils as possible sources, while a minority (4%) expressed concerns about transmission through footwear and clothing. Airborne transmission was acknowledged by 13%. Surprisingly, 43% were unaware of the sources of disease transmission.

#### 3.3.3 Handling a Disease Situation

**FIGURE 3:**
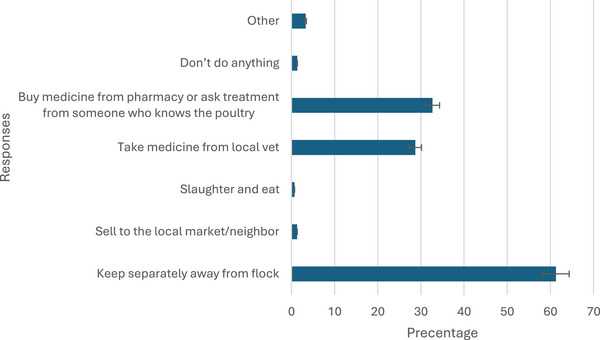
What they do for sick birds.

The most common management practice for sick birds, reported by 61% of respondents, was isolating them from the rest of the flock. However, a small percentage (1%) mentioned selling sick birds to local markets or neighbours and 1% reported slaughtering and consuming sick birds (Figure 3).

The predominant practice for disposing of dead birds, reported by 96% of respondents, was burying the carcasses. Only a few respondents (2%) mentioned throwing dead birds away allowing them to be consumed by other animals. However, respondents mentioned burning the carcasses, and there were no reported alternative practices for the disposal of dead birds.

#### 3.3.4 Awareness Regarding Impact on Humans

Regarding the potential impact of poultry pathogens on humans, almost a third (29%) recognized that poultry pathogens could affect humans and cause diseases. Conversely, 21% believed poultry pathogens could not impact humans in terms of causing diseases, while 49% were uncertain.

#### 3.3.5 Knowledge and Practices Regarding Vaccination

**FIGURE 4:**
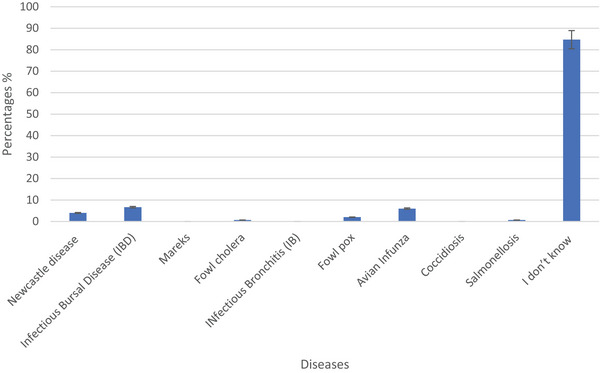
Which diseases could be effectively prevented through vaccination.

Approximately 53% of respondents were aware that vaccines could prevent poultry diseases. When asked about their knowledge regarding diseases that can be effectively prevented with vaccines, the respondents provided varying responses. Less than a tenth of the participants (8%) were aware that vaccines could effectively prevent viral diseases (Table 2). Regarding diseases preventable with vaccines, a significant majority of respondents (84.67%) admitted to not knowing which diseases could be effectively prevented through vaccination. Among those who provided specific answers, 4%, 7% and 6% of the respondents answered that Newcastle disease, infectious bursal disease (IBD) and avian influenza can be prevented with vaccines respectively (Figure 4).

**TABLE 2:** Their knowledge regarding diseases that can be effectively prevented with vaccines.

Response	Frequency	Percentage
Bacteria only	4	2.7
Virus only	12	8
I do not know	113	75.3
Both viruses and bacteria	21	14

A majority (80%) had never vaccinated their birds, while 18% reported having vaccinated their birds at least once. However, 2% were unsure or could not remember vaccination status of their birds.

### 3.4 Constraints and Challenges in Family Poultry Farming

**FIGURE 5:**
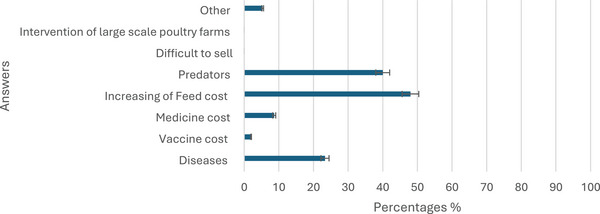
Problems faced in poultry production.

Family poultry keepers in the study area face several constraints that limit the expansion of their farms. These include land limitations (36%), limited technical knowledge (3%), financial constraints (48%), difficulties in obtaining bank loans (5%) and unspecified challenges (11%). Diseases were a concern for 23% of respondents, posing economic and productivity challenges. The increasing cost of feed was a significant problem for 48% of respondents, impacting profitability. Predators were identified as a challenge by 40% of participants, leading to losses (Figure 5).

Most respondents (59%) reported neutral neighbour attitudes towards their family poultry farming, while 23% faced opposition due to concerns like smell (20%) and damage done to neighbouring cultivations (69%) by the extensively managed chickens.

A significant proportion of respondents (80%) expressed interest in farm expansion, with 87% believing it would lead to increased product sales; however, 9% were uncertain about the impact of farm expansion on product sales.

### 3.5 Information Exposure and Preferred Learning Methods in Poultry Management

Approximately 54% of respondents had prior exposure to poultry management information, while 46% did not. The majority (90%) of respondents expressed a strong desire to enhance their knowledge on poultry management. They indicated a preference for learning through various methods, including leaflets, booklets, videos and workshops.

## 4 Discussion

The demographic characteristics of the respondents revealed important aspects of the family poultry farming community in the surveyed area. The survey highlighted the predominance of female respondents (68%), emphasizing their vital role in household food security and income generation through family poultry farming. Similar to this finding, women play a pivotal role in family poultry farming on a global scale, especially in a rural setting (Kumar, Dahiya, and Ratwan 2021). Further, a study conducted in West Bengal revealed that engaging in family poultry farming has empowered women both economically and socially, elevating their status within the family and community (Gupta et al. 2021). Given that 65% of respondents completed up to ordinary‐level education, customized training programmes could significantly improve poultry management practices and overall farm productivity.

The involvement of companion animals, livestock and other bird species alongside chicken highlights integrated farming approaches. Similarly, family poultry rearing plays an essential role in integrated farming systems worldwide (Paramesh et al. 2019). Our study found that 13.3% raised other bird species, such as pigeons, alongside chicken. Similarly, a study of backyard poultry flocks in Morocco documented a diverse range of species: 83% chickens, 7% guinea fowls, 6% pigeons, 2% turkeys and 1% ornamental birds such as peacocks, geese and ducks (Fagrach et al. 2023).

The reliance on wells for water sources and the use of both commercial feed and family leftovers for nutrition highlight resourceful farming practices. In our study, 57% of farmers fed chickens both commercial feed and leftover family food, 35% allowed chickens to scavenge and 20% provided only commercial feed. Compared to another study on poultry revealed that backyard birds primarily fed on what they find in their environment such as seeds, insects, and worms while scavenging (Fagrach et al. 2023). However, 96% of flock owners supplement their diet, commonly with barley (94%) and stale bread (81%), followed by kitchen waste (65%), wheat bran (63%) and wheat (45%) (Fagrach et al. 2023).

The findings underscore an interplay between poultry farming and household income dynamics. While 64% of households aimed to supplement their income through poultry production, only 3% relied solely on it as their primary source of income. This suggests that for many, poultry farming serves as a supplementary rather than a primary income source. Interestingly, the study revealed that poultry income disproportionately benefits poorer households, with the lowest quintile generating 44%, compared to 15% for the wealthiest. For many low‐income families, poultry income serves as their primary livestock cash source (Birhanu et al. 2023).

The findings regarding biosecurity practices among the respondents were important. Among those familiar with the term ‘biosecurity’, it is concerning that none exhibited good biosecurity practices. A majority of this group, 76%, demonstrated poor practices, highlighting a substantial gap in understanding and implementation of biosecurity measures. Only 24% showed moderate practices, indicating room for improvement in raising awareness and encouraging better biosecurity practices in this subgroup. Conversely, among respondents unfamiliar with the term ‘biosecurity’, while the majority still had poor practices (73%), a small percentage (4%) adhered to good biosecurity measures, and 23% practised at a moderate level. This suggests that practical knowledge and practices exist, even in the absence of formal awareness, emphasizing the need for education and training programmes to bridge this knowledge gap. The study underscores the necessity of targeted interventions to enhance biosecurity practices across both groups. It is imperative to categorize the forms of knowledge displayed in biosecurity to better understand their origins–whether they are primarily indigenous or scientific. This distinction is crucial for effective knowledge co‐production among the locals. A recent study conducted among family poultry keepers in Bangladesh emphasized the significance of awareness programmes on biosecurity. The study found that familiarity with the term ‘biosecurity’ was significantly higher among keepers who had received training programmes on the topic compared to those who had not. This highlights the effectiveness of education and training initiatives in enhancing biosecurity practices and overall disease management in family poultry farming (Khalil et al. 2023). And also, the surge in backyard farming during the COVID‐19 pandemic in Cambodia, characterized by limited biosecurity measures, raises concerns about the heightened risk of zoonotic disease transmission (Hyder et al. 2023).

The current study findings highlight a significant gap in knowledge among poultry keepers concerning poultry pathogens. Specifically, the study notes that only a minority (21%) recognized viruses as causes of diseases. Concerningly, a notable proportion (51%) was uncertain about whether disease‐causing microorganisms could spread among chickens, indicating a lack of clarity in this area. The findings regarding the awareness of potential poultry pathogen transmission to humans are indicative of the knowledge gaps among respondents. While almost a third (29%) recognized that poultry pathogens can lead to diseases in humans, an even more significant proportion of farmers (49%) expressed uncertainty about this matter. This variability underscores the need for targeted educational campaigns to enhance understanding about zoonotic diseases. Raising awareness about the potential risks associated with poultry pathogens is essential, as it can lead to more proactive biosecurity measures, reducing the chances of zoonotic spillover and improving public health outcomes. A study conducted in Nepal among commercial poultry farmers revealed that farmers had more knowledge about poultry avian influenza symptoms compared to human avian influenza symptoms (Lambrou et al. 2020). Another study conducted in Tulsipur sub‐metropolitan city, Nepal, aimed to assess the KAP regarding zoonotic diseases among smallholder livestock owners. A significant finding was the positive association between farmers' educational levels and their knowledge of zoonoses, with those having lower educational backgrounds demonstrating less awareness of zoonotic diseases (Niraulaa, Sharmaa, and Dahalb 2021).

In addition to identifying gaps in knowledge about poultry pathogens, the study also observed that self‐administering medication (33%) was common among studied keepers. The behaviour of self‐administering drugs without proper medical guidance has been reported globally (Kiambi et al. 2021; Dyar et al. 2020; Subedi et al. 2023). For example, a study conducted in Kenya sheds light on the challenges related to antimicrobial use in small‐scale layer farms. The research revealed that commonly used antibiotics were often labelled for various purposes, including prophylactic use, growth promotion and egg production improvement. Additionally, the study highlighted that antimicrobial use was frequently driven by the presence of diseases or disease symptoms that could potentially be managed more effectively through infection prevention measures (Kiambi et al. 2021).

While more than half of sampled farmers (53%) were aware that vaccines could prevent poultry diseases, there was limited knowledge about specific diseases that could be prevented. A majority (80%) had never vaccinated their birds, suggesting missed opportunities for disease prevention through vaccination. Similarly, a study conducted in Nigeria reported a relatively low vaccination rate against poultry coccidiosis, with only 7% of farmers using vaccination as a preventive measure (Adeyemi et al. 2023).

Family poultry farmers in the survey encounter various constraints, including limited land availability, financial constraints and difficulties in accessing bank loans. Diseases, high cost of vaccines and medicines and rising feed expenses pose challenges. Predators are also a significant concern. These challenges and constraints were not unique to the studied group, as similar issues and limitations have been reported among poultry keepers in other countries as well (Rajkumar et al. 2021; Chaiban et al. 2020). Notably, a substantial 80% expressed interest in farm expansion, with a majority believing it could boost product sales, demonstrating the potential for growth in the family poultry sector.

A significant portion of respondents lacked prior exposure to poultry management information. However, the strong interest in learning and preferred methods highlights opportunities for education and training in this sector.

The study has several limitations, including potential sampling bias, which may affect the generalizability of the findings. Additionally, the lack of inferential statistics means that the recommendations are based primarily on descriptive results, which is not ideal for drawing definitive conclusions. The absence of qualitative data and the failure to account for external factors further limit the robustness of the findings. These limitations should be considered when interpreting the results and applying the recommendations.

## 5 Conclusion

The study highlights the significant knowledge deficits among family poultry keepers concerning poultry diseases, their causes, transmission pathways and biosecurity measures. This information gap poses a potential risk not only to the poultry sector but also to human health. Urgent government and policy interventions are imperative to facilitate farmer access to vaccinations and educational programmes. These initiatives can empower farmers with the knowledge and practices needed for disease control and effective biosecurity, ultimately benefiting the livelihoods of these communities and mitigating the risk of zoonotic disease transmission to the broader population. Emphasis should be placed on those who are familiar with the term ‘biosecurity’ but still practise it poorly to ensure that interventions are appropriately tailored to their specific knowledge base and needs. Since many farmers primarily rear poultry for egg production, it is recommended to encourage them to consider poultry as a source of meat as well.

## Author Contributions

**Umayangana Pujani Gunasekara**: data curation, formal analysis, methodology, software, validation, visualization, writing–original draft. **Anil Wasantha Kalupahana**: conceptualization, investigation, project administration. **Yasodhara Deepachandi Gunasekara**: data curation, formal analysis, methodology, writing–review and editing. **Ayona Silva‐Fletcher**: conceptualization, funding acquisition, project administration, supervision, writing–review and editing. **Ruwani Sagarika Kalupahana**: conceptualization, data curation, investigation, methodology, project administration, supervision, writing–original draft, writing–review and editing.

## Consent

The authors confirm that the ethical policies of the journal, as noted on the journal's author guidelines page, have been adhered to and the appropriate ethical review committee approval has been received. The US National Research Council's guidelines for the Care and Use of Laboratory Animals were followed.

## Conflicts of Interest

The authors declare no conflicts of interest.

## Peer Review

The peer review history for this article is available at https://publons.com/publon/10.1002/vms3.70214.

## Data Availability

The data that support the findings of this study are available on request from the corresponding author. The data are not publicly available due to privacy or ethical restrictions
